# PTAA‐Based Perovskite Photovoltaics Catching up: Ionic Liquid Engineering‐Assisted Crystallization Through Sequential Deposition

**DOI:** 10.1002/advs.202414515

**Published:** 2025-02-20

**Authors:** Yongjun Li, Fei Wang, Qiannan Li, Baolei Tang, Yonggui Sun, Taomiao Wang, Xiao Liang, Jing Ma, Xianfang Zhou, Fan Zhang, Xing'ao Li, Yao Tong, Ruiyuan Hu, Mingjian Yuan, Tom Wu, Annie Ng, Hanlin Hu

**Affiliations:** ^1^ Hoffmann Institute of Advanced Materials Shenzhen Polytechnic University 7098 Liuxian Boulevard Shenzhen 518055 China; ^2^ Jiangsu Provincial Engineering Research Center of Low‐Dimensional Physics and New Energy & School of Science Key Laboratory for Organic Electronics and Information Displays & Institute of Advanced Materials (IAM) Jiangsu National Synergistic Innovation Center for Advanced Materials (SICAM) Nanjing University of Posts and Telecommunications Nanjing 210023 China; ^3^ State Key Laboratory of Advanced Technology for Materials Synthesis and Processing School of Materials Science and Engineering Wuhan University of Technology Wuhan 430070 China; ^4^ Medical Intelligence and Innovation Academy Southern University of Science and Technology Hospital Shenzhen 518055 China; ^5^ College of Chemistry Nankai University Tianjin 300071 China; ^6^ Department of Applied Physics The Hong Kong Polytechnic University Kowloon Hong Kong; ^7^ Department of Electrical and Computer Engineering, School of Engineering and Digital Sciences Nazarbayev University 53 Kabanbay Batyr Avenue Astana 010000 Kazakhstan

**Keywords:** crystallinity, ionic liquid, perovskite solar cells, p‐i‐n, PTAA

## Abstract

PTAA as a widely studied polymeric hole transporting material, has garnered significant attention due to its outstanding thermal and chemical stability. However, the performance of PTAA‐based p‐i‐n devices is shown to lag behind counterpart utilizing oxides or SAMs. In this study, the ionic liquid, 1‐ethyl‐3‐methylimidazolium formate (EMIMCOOH), is innovatively introduced into the lead iodide (PbI_2_) precursor solution, resulting in a more pronounced mesoporous PbI_2_ film with expended pore‐size and denser pores. This enhancement is attributed to the coordination bond between the ─C═O group in EMIMCOOH and Pb^2+^. This intensified mesoporous morphology not only facilities the reaction between PbI_2_ and the organic layer, but also promotes the PbI_2_ conversion into perovskite material. Importantly, the incorporation of EMIMCOOH slows down the perovskite conversion process, increasing perovskite domain size and suppressed Pb^0^ trap density, resulting in a uniform perovskite layer with enhanced charge transport properties, as evidenced by the conducting atomic force microscope (c‐AFM) results. As a result, the incorporation of EMIMCOOH yields a power conversion efficiency (PCE) of 24.10% and a high fill factor exceeding 85%. Notably, the PCE of the EMIMCOOH‐modified device can still maintain 86% of the initial value after 1500 h at 25 °C in an N_2_ atmosphere.

## Graphical abstract

In the investigation, they introduce the ionic liquid 1‐ethyl‐3‐methylimidazolium formate (EMIMCOOH) into the lead iodide (PbI_2_) precursor solution, resulting in a mesoporous PbI_2_ film with larger pore sizes, which enhanced the conversion of PbI_2_ into perovskite material. The power conversion efficiency (PCE) of EMIMCOOH‐modified PTAA‐based inverted PSCs achieved a notable 24.10%, with a fill factor exceeding 85%.

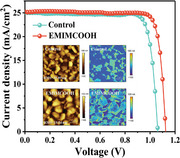

## 1 Introduction

Perovskite solar cells (PSCs) have drawn remarkable attention due to their excellent photoelectric properties, such as tunable band gap, high absorption coefficient, exceptional high carrier mobilities, diffusion length, and so on.^[^ ^1^, ^2^, ^3^, ^4^ ^]^ To date, the power conversion efficiencies of PSCs have rapidly increased to a certified value of 26.7% in both regular (n‐i‐p) and inverted (p‐i‐n) architectures, positioning them as formidable contenders for dominating the next generation of the photovoltaic industry, comparable to commercially available monocrystalline silicon solar cells.^[^^]^It is worth noting that inverted PSCs possessed the merits of low‐temperature processibility, negligible hysteresis, and excellent stability catering to fabricating tandem solar cells. Poly[bis(4‐phenyl)‐(2,4,6‐trimethylphenyl)amine] (PTAA) is one of the most widely used and effective hole transport materials for inverted PSCs due to its facile preparation process, good stability, and relatively matched energy levels with the perovskite.^[^ ^10^, ^11^, ^12^ ^]^ Currently, the one‐step deposition process assisted by antisolvent is the most widely employed method to prepare high‐performance inverted PSCs, but it is difficult to ensure operational robustness and replicability owing to the stringent control required during antisolvent dripping.^[^ ^13^ ^]^ However, the two‐step sequential deposition method exhibits a facile fabrication process and exceptional repeatability,^[^ ^14^, ^15^ ^]^ and can effectively control the growth of perovskite crystals, morphology, and thickness of the films by management of the first‐step deposition process.^[^ ^16^ ^]^

An excess amount of lead iodide (PbI_2_) is prone to locate in the perovskite films via two‐step sequential deposition due to the incomplete conversion of PbI_2_ and degradation of perovskite films in the PSCs devices prepared.^[^ ^17^, ^18^ ^]^ It has been confirmed that the excess PbI_2_ in perovskite films is beneficial for reducing the concentration of halide vacancies, regulating the crystallization of perovskite, and eliminating the hysteresis effect of devices, thereby improving the PCE of devices.^[^ ^19^ ^]^ However, the distribution of excess PbI_2_ in perovskite films is difficult to control, which may lead to the PbI_2_ residue random aggregation in the perovskite films or on the perovskite upper and lower surfaces.^[^ ^20^, ^21^, ^22^ ^]^ The excessive aggregation of PbI_2_ significantly deteriorates the quality of perovskite and hinders charge transfer at the interface. Besides, the excess PbI_2_ within perovskite films will decompose into Pb^0^ and I_2_ under illumination, which is widely considered as one of the most detrimental factors for the deterioration of PSCs and subsequently compromised device stability.^[^ ^17^, ^23^ ^]^

Recently, incorporating different types of additives into PbI_2_ precursor solutions to eliminate the residua PbI_2_ in perovskite films has been one of the popular strategies to improve the stability and efficiency of PSCs.^[^ ^22^, ^23^, ^24^, ^25^, ^26^, ^27^, ^28^, ^29^ ^]^ Zhao et al. converted excess residual PbI_2_ into an inactive secondary‐phase (PbI_2_)_2_RbCl compound by RbCl incorporating, which effectively stabilizes the perovskite phase.^[^ ^31^ ^]^ Liu et al. introduced ammonium chloride (QAH) additive into the PbI_2_ precursor to induce the formation of a porous structure in the PbI_2_ film.^[^ ^32^ ^]^ The modulated PbI_2_ film facilitated the infiltration of organic salts and promoted a more thorough interaction between PbI_2_ and the organic salt promoted the transformation of PbI_2_ into the perovskite phase, forming high‐quality perovskite. Wang and his coworkers introduced 4‐Fluorobenzamide (FBAD) additives into the precursor FAI to delay the interaction between PbI_2_ and organic salts, which efficiently decreased the residual amount of PbI_2_ and modulated the crystallization process of perovskite to obtain high‐quality perovskite film.^[^ ^30^ ^]^

Ionic liquids (ILs), characterized by low volatility, tunable physicochemical, high carrier mobility, excellent conductivity, and superior thermal and electrochemical stability, have become ideal candidates as additives for the field of perovskite photovoltaics.^[^ ^33^, ^34^, ^35^ ^]^ These features play multiple functions for efficient and stable PSCs, including the management of perovskite crystallization, interface modification, defect passivation, and the provision of innovative alternatives to conventional materials.^[^ ^36^, ^37^, ^38^ ^]^ For example, the Sai Bai team incorporated the ionic liquid BMIMBF_4_ into perovskite films, resulting in improved the device efficiency and long‐term stability. Under continuous simulated full‐spectrum sunlight at temperatures between 70 and 75 °C for over 1800 h, the performance of the most stable encapsulated device decreased by only ≈5%.^[^ ^39^ ^]^ In addition, Yen‐Hung Lin and his team successfully mitigated the generation of impurity phases caused by component separation by incorporating piperidine‐based ionic compounds into the formamidinium‐cesium lead‐trihalide perovskites. This method effectively adjusted the bandgap, making the material highly suitable for silicon‐based perovskite tandem solar cells, thereby enhancing the open‐circuit voltage and PCE, and optimizing the material's overall performance.^[^ ^40^ ^]^ In this work, we introduce a functional IL additive of 1‐ethy‐3‐methylimidazolium formate(EMIMCOOH) into the PbI_2_ precursor solution to fabricate PTAA‐based inverted PSCs by the two‐step sequential deposition method. The uniform porous nature of PbI_2_ facilitates the permeation of organic salts into the PbI_2_ film, leading to the formation of flat and dense, perovskite films. Moreover, the full reaction between organic salts and PbI_2_ can mitigate device instability resulting from excessive PbI_2_ residue. The IL additive can interact with PbI_2_ to retard the crystallization process of perovskite, resulting in the formation of large particle sizes, few defects, and more uniform high‐quality perovskite films. As a result, the optimized device, based on EMIMCOOH‐modulated perovskite, achieves a significantly higher efficiency of 24.10% compared to the control device's efficiency of 22.18%, and notable enhancements are observed in both the *V_OC_* and fill factor (*FF*). Moreover, the unencapsulated devices show excellent stability, maintaining 90% of their initial efficiency after 1500 h in an environment at 25 °C under a N_2_ atmosphere. This strategy of preparing uniformly porous PbI_2_ to enhance the diffusion of organic salts, thereby proficiently managing residual PbI_2_ and improving perovskite crystal quality and stability, holds great potential for facilitating the commercialization of PSCs.

## 2 Results and Discussion

**Figure 1:**
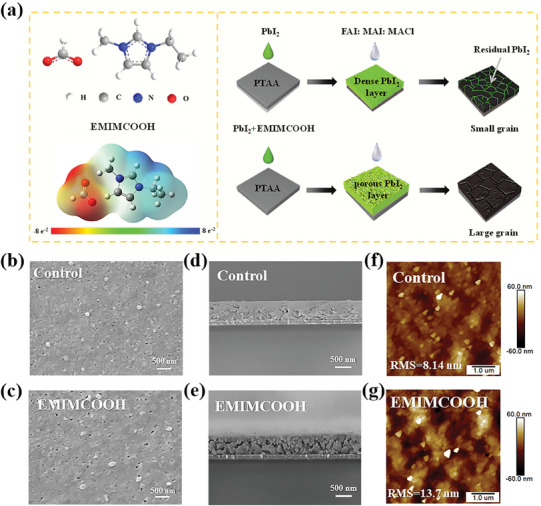
a) Chemical structure and surface electrostatic potential of the IL EMIMCOOH, and schematic illustration of the process for producing perovskite films using a two‐step sequential deposition technique. b,c) Top‐view SEM images, d,e) cross‐sectional SEM images, and f,g) AFM images of the PbI_2_ film and the PbI_2_ + EMIMCOOH film.

PTAA as a widely used polymeric hole transporting material with outstanding chemical and thermal stability has attracted huge research interest in perovskite community. However, the performance of PTAA‐based p‐i‐n devices has lagged behind than the counterpart of small molecules or oxides.^[^ ^28^, ^41^, ^42^ ^]^ In this study, a novel ionic liquid (EMIMCOOH) was introduced into the PbI_2_ precursor solution as an additive to manipulate the perovskite film formation process using a two‐step sequential deposition method. The chemical structure and surface electrostatic potential of the EMIMCOOH are illustrated in **Figure** **1**a. The electrostatic potential surface map of EMIMCOOH shows that the HCOO⁻ substituent acts as a strong negative charge center (indicated by the red and yellow areas), suggesting that HCOO⁻ in EMIMCOOH serves as a potential binding site for perovskite. Furthermore, we conducted conductivity experiments to assess the impact of introducing EMIMCOOH on the charge transfer performance of perovskite film and PSC. As shown in Figure S1 (Supporting Information), the pure EMIMCOOH film exhibited evident conductivity, in contrast to the pristine glass, which is insulating. This indicates that the incorporation of EMIMCOOH does not significantly hinder the charge transfer capability of the perovskite film or the corresponding PSC.^[^ ^43^, ^44^, ^45^ ^]^ Detailed schematic diagrams of fabrication procedure for the perovskite films and PTAA‐based inverted PSCs can be found in Figure 1a and Figure S2 (Supporting Information). The quality of the perovskite film deposition method was largely dependent on the morphology and crystallinity of the PbI_2_ film. Energy Dispersive Spectroscopy (EDS) mapping of both control and EMIMCOOH‐modified PbI_2_ films confirmed the existence of EMIMCOOH in PbI_2_ films (Figures S3 and S4, Supporting Information).^[^ ^46^ ^]^ To investigate the effect of EIMICOOH additive on PbI_2_ films, the surface morphology of PbI_2_ with or without EIMICOOH modification was observed by scanning electron microscopy (SEM), as shown in Figure 1b,e. Control PbI_2_ film exhibited a compact morphology with small grain size and less porous structures. The dense PbI_2_ film presented a challenge in achieving a complete reaction with the mixed organic salt solution, resulting in residual PbI_2_ persisting within the grain boundaries of the perovskite, ultimately impacting device performance.^[^ ^28^ ^]^

After introducing EMIMCOOH, the PbI_2_ film exhibited higher porosity. This uniform mesoporous structure of the EMIMCOOH‐modified PbI_2_ films was further confirmed by the cross‐section SEM images (Figure 1d,e), which was beneficial for the penetration of organic salts, reacting with PbI_2,_ and triggering the crystal growth.^[^ ^47^, ^48^ ^]^ Additionally, atomic force microscope (AFM) images were acquired to further investigate the influence of the IL EMIMCOOH on the morphology of PbI_2_ (Figure 1f,g; Figure S5, Supporting Information). The root‐mean‐square (RMS) roughness of the control PbI_2_ film (8.14 nm) was smaller compared to that of the EMIMCOOH‐modified PbI_2_ film (13.7 nm), further revealing the morphological change in the PbI_2_ film. The result was consistent with the SEM image.

**Figure 2:**
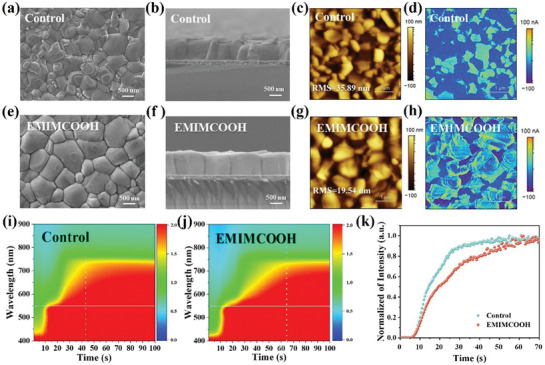
Top‐view and cross‐sectional SEM images of the a,b) control and e,f) EMIMCOOH‐modified perovskite films. AFM and c‐AFM images of the c,d) control and g,h) EMIMCOOH‐modified perovskite films on PTAA‐coated ITO glass. In situ UV–vis absorption spectroscopy of i) control and j) EMIMCOOH‐modified perovskite film during annealing. k) The intensity of the in situ absorption peak during annealing was compared between the control perovskite film and the perovskite film with EMIMCOOH.

To further confirm the influence of the EMIMCOOH on perovskite crystallization and stability, the surface morphology of the control and EMIMCOOH‐modified perovskite films was investigated by top‐view and cross‐sectional SEM images. **Figure** **2**a,e showed the top‐view SEM images of control and EMIMCOOH‐modified perovskite films. Compared to the control perovskite films, the EMIMCOOH‐modified perovskite films exhibited a flatter morphology with larger grain sizes, indicating a significant improvement in film quality of perovskites through EMIMCOOH modification. The EMIMCOOH‐modified perovskite film displayed larger average grain size of 1150 nm than that of 850 nm for the control film (Figure S6, Supporting Information). Notably, the morphology analysis of the control perovskite films revealed the presence of numerous white phases, previously identified as unreacted and residues PbI_2_.^[^ ^47^ ^]^ The presence of residues PbI_2_ impeded the transport and collection of charge carriers, leading to nonradiative recombination and subsequent energy loss and performance degradation in the devices. However, the residual PbI_2_ was significantly reduced on the surface of perovskite films after the introducing IL EMIMCOOH. This may be attributed to the mesoporous structure of the EMIMCOOH‐modified PbI_2_ film facilitated the diffusion of organic salts, promoting the conversion process of PbI_2_ into the perovskite and providing the necessary space for perovskite crystal growth.^[^ ^49^, ^50^, ^51^ ^]^ Cross‐sectional SEM analysis further confirmed the phenomenon of grain enlargement and a significant reduction in residual PbI_2_ in the perovskite film after the EMIMCOOH modification (Figure 2b,f). The AFM images were acquired to further investigate the influence of the EMIMCOOH on the morphology of resulted perovskite thin films. The IL EMIMCOOH modification could effectively enhance the crystal grain size of perovskite and decrease the surface roughness of perovskite film (Figure 2c,g), which was consistent with the above SEM results. The conductive atomic force microscopy (c‐AFM) images in Figure 2d,h revealed that the EMIMCOOH‐modified perovskite film exhibited a more pronounced enhancement in the current signal compared to the control film. The noticeable current change indicated that modifying with the EMIMCOOH could significantly enhance the conductivity of the film.^[^ ^52^ ^]^ This improvement was attributed to the increased grain size, reduced grain boundary, and passivation of perovskite defects by the EMIMCOOH.

To further investigate the impact of the EMIMCOOH on regulating perovskite grain crystal conversion process, we utilized in situ UV–vis absorption measurements to dynamically monitor the conversion process under thermal treatment of both the control and EMIMCOOH‐modified perovskite films. The spectral intensity exhibited a gradual increase within the range of 400–900 nm during the thermal annealing stage, indicating the progressive formation of the black perovskite phase. Finally, the absorption spectral intensity reached a peak and remained stable, indicating the complete conversion of the perovskite, as illustrated in Figure 2i–k. The perovskite conversion in the control perovskite films was fully completed within 43 s, along with a simultaneous broadening and enhancement of the absorption spectrum. In contrast, the EMIMCOOH‐modified perovskite exhibited a longer conversion time, stabilizing at 68 s. This result suggested that the introduction of the IL EMIMCOOH significantly prolonged the perovskite conversion process, thereby promoting the development of high‐quality perovskite films with larger grains and diminished residual PbI_2_. The prolonged conversion process for EMIMCOOH‐modified perovskite films may be attributed to the strong interaction between PbI_2_ and EMIMCOOH, resulting in an increased energy barrier for nucleation and growth of perovskite.^[^ ^53^, ^54^, ^55^ ^]^

**Figure 3:**
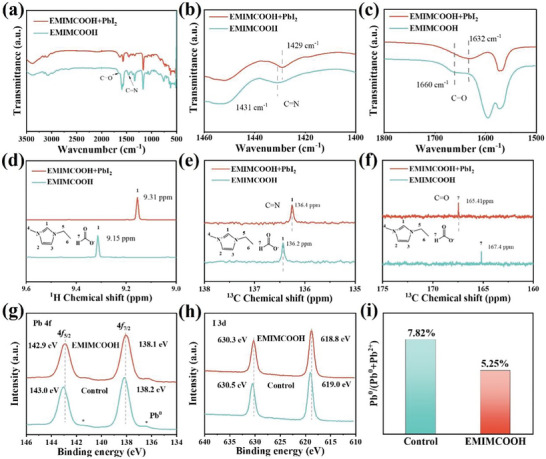
a‐c) FTIR spectra of EMIMCOOH and EMIMCOOH + PbI_2_ solutions. d) ^1^H NMR and e,f) ^13^C NMR spectra of EMIMCOOH and EMIMCOOH + PbI_2_ solutions. g) Pb 4f XPS spectra of perovskite films with or without EMIMCOOH. h) I 3d XPS spectra of perovskite films with or without EMIMCOOH. i) The statistical proportions of Pb^0^ in the perovskite films from the results of high‐resolution XPS spectrum for Pb 4f.

To investigate the chemical interaction between PbI_2_ and EMIMCOOH, we performed the Fourier transform infrared spectra (FTIR) on both EMIMCOOH and EMIMCOOH‐PbI_2_ samples, as presented in **Figure** **3**a–c. EMIMCOOH displayed a distinct vibration peak of C═N at 1431 cm^−1^ and asymmetric stretching vibration peaks of C═O at 1660 cm^−1^. After the addition of PbI_2_, the C═N peak shifted to a lower binding energy from 1431 to 1429 cm^−1^. This shift demonstrated the interaction between the EMIM^+^ cation and PbI_2_,^[^ ^56^ ^]^ delaying the reaction process between mixed organic salts and PbI_2_ and prolonging perovskite crystal growing time. At the same time, the asymmetric and symmetric stretching vibration peaks of C═O in EMIMCOOH interact with PbI_2_, causing the corresponding peaks to shift to 1632 cm^−1^. This phenomenon proved the strong interaction between acetate carbonyl and Pb.^2+[^ ^57^ ^]^ The interaction between the anions and cations of EMIMCOOH and PbI_2_ played a pivotal role in the formation of a homogeneous porous PbI_2_ layer. This strong interaction could then passivate the grain boundary and surface uncoordinated Pb^2+^of the perovskite film.^[^ ^48^ ^]^

To further verify the above conclusion, the nuclear magnetic resonance (NMR) spectra of EMIMCOOH and EMIMCOOH‐PbI_2_ samples were collected and were shown in Figure 3d and Figure S7 (Supporting Information). The addition of PbI_2_ to the EMIMCOOH solution resulted in a shift of the resonance peak of the imidazole group from 9.31 ppm to 9.15 ppm. This shift was attributed to the coordination between the C═N group in the imidazole group and the uncoordinated Pb^2+^. Furthermore, the ^13^C NMR analysis revealed a slight shift in the chemical shift of the C═N resonance peak from 136.4 ppm to 136.2 ppm (Figure 3e,f; Figure S8, Supporting Information), providing supplementary evidence for the interaction between the imidazole group and PbI_2_.^[^ ^46^ ^]^ Notably, the chemical shift of the C═O group in the IL EMIMCOOH changed from 165.1 ppm to 167.4 ppm. To confirm the chemical interaction between EMIMCOOH and perovskite material, we investigated the surface chemical state of perovskite thin films by X‐ray photoelectron spectroscopy (XPS). For the control perovskite film, the XPS peaks at 143.0 and 138.2 eV were identified as Pb 4f_5/2_ and Pb 4f_7/2_, respectively (Figure 3g; Figure S9, Supporting Information). For the IL EMIMCOOH‐modified perovskite film, the XPS peaks at 142.9 and 138.1 eV were identified as Pb 4f_5/2_ and Pb 4f_7/2_, respectively. In comparison with the control perovskite film, these peaks in the EMIMCOOH‐modified perovskite films shifted to lower binding energies, indicating an increase in the electron cloud density near Pb^2+^ in the perovskite film treated with EMIMCOOH. This shift was attributed to the anion HCOO^‐^ functioning as an electron donor, supplying electrons to the Pb atom and establishing coordination bonds with coordinated Pb^2+^ ions through Lewis acid‐base coordination.^[^ ^58^, ^59^, ^60^ ^]^ This effectively reduced uncoordinated Pb^2+^ defects in the perovskite, thereby inhibiting Pb^2+^ from being reduced to Pb^0^ and improving the stability of the perovskite film. Compared to the core levels of I 3d (619.0 and 630.5 eV) in the control film, the binding energies of the I 3d core levels (618.8 and 630.3 eV) in the EMIMCOOH‐modified perovskite film was slightly shifted to lower values, indicating a chemical interaction between the C═O in HCOO^−^ and I.^[^ ^60^ ^]^ Additionally, Pb^0^ exhibited a decreasing trend in both the control and EMIMCOOH‐modified perovskite film (Figure 3i), which was important evidence for explaining the reduction of residual PbI_2_.

**Figure 4:**
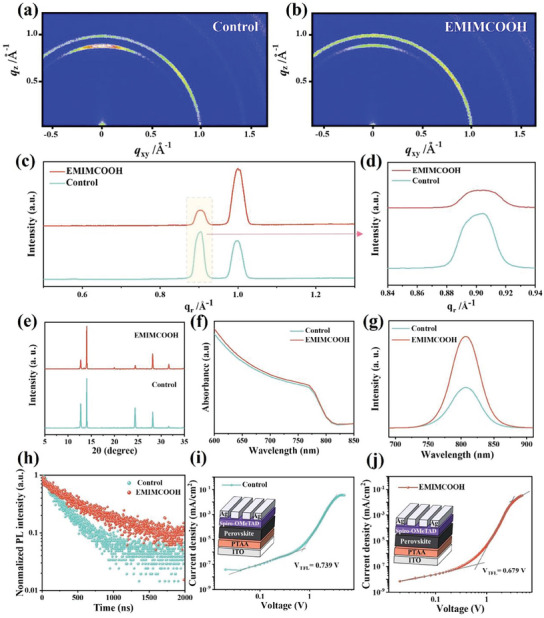
The 2D GIWAXS spectra of a) control and b) EMIMCOOH‐modified perovskite film. c,d) The integration of both control and EMIMCOOH‐modified perovskite 2D GIWAXS patterns in‐plane. e) XRD patterns of control and EMIMCOOH‐modified perovskite films. f) UV–vis absorbance spectra of control and EMIMCOOH‐modified perovskite films. g) Steady‐state photoluminescence (PL) spectra, and h) time‐resolved photoluminescence of the control and EMIMCOOH‐modified perovskite films, respectively. i,j) Dark *I*–*V* curves of the control and EMIMCOOH‐modified perovskite films with a structure of ITO/PTAA/perovskite/Spiro‐OMeTAD/Ag.

To quantitatively determine the residual amount of PbI_2_ in perovskite films, we performed grazing incidence wide‐angle X‐ray scattering (GIWAXS) measurement for both the control and EMIMCOOH‐modified perovskite films. Obvious scattering rings can be observed at *q* = 1.0 Å⁻¹ (where *q* = (4πsinθ) / λ), corresponding to the perovskite crystal (110) plane of the perovskite film with or without IL EMIMCOOH modification, as shown in **Figure** **4**a,b. Compared with the control perovskite film, the scattering peak intensity of the (110) crystal plane of the perovskite film modified by EMIMCOOH increased. Additionally, the peak corresponding to *q* = 0.9 Å^−1^ was identified as PbI_2_, and its intensity was significantly reduced.^[^ ^35^ ^]^ A 2D GIWAXS integral image was created, as shown in Figure 4c,d. The 2D GIWAXS integral image of the EMIMCOOH‐modified perovskite film showed a significant decrease in the peak intensity of PbI_2_, while the peak intensity of the perovskite (110) crystal plane increased. This was due to the interaction between EMIMCOOH and PbI_2_, which altered the morphology of the PbI_2_ layer, creating a uniform and porous structure. This structure facilitated the wide diffusion of mixed organic salts, ensuring the complete conversion of PbI_2_ into perovskite. Consequently, this process reduced the residual amount of PbI_2_ in the perovskite film, enhancing the stability of perovskite films. To further verify the effect of EMIMCOOH on the crystallinity of perovskite crystals, we collected X‐ray diffraction (XRD) patterns of perovskite films without EMIMCOOH treatment and those treated with EMIMCOOH. The results (Figure 4e; Figure S10, Supporting Information), consistent with the GIWAXS spectra, showed that the peak intensity associated with PbI_2_ initially decreased with increasing EMIMCOOH concentration, while the peak intensity related to perovskite increased. Too high concentration of EMIMCOOH also led to the decrease of the peak intensity corresponding to the perovskite crystals. Thus, an optimal concentration of EMIMCOOH doping was essential for improving the crystal quality of the perovskite film. Further, by analyzing the results of the XRD, we calculated the full width at half maxima (FWHM), and the results are shown in the Figure S10 (Supporting Information). The FWHM of the perovskite film modified with EMIMCOOH is significantly smaller than that of the control group, indicating an improvement in crystallinity. This result is consistent with the observations from SEM images. The UV–vis absorption test was then measured to indicate the identical absorption threshold for control and EMIMCOOH‐modified perovskite film, further proving the maintenance of lattice consistent with the XRD and GIWAXS consequences(Figure 4f).^[^ ^61^ ^]^

To investigate the key optoelectronic properties of perovskite film, we conducted the steady‐state photoluminescence (SSPL) spectra tests, as illustrated in Figure 4g. Compared with the control perovskite film, the photoluminescence (PL) peak intensity of the perovskite film treated with EMIMCOOH was significantly increased, indicating that the perovskite crystal defects in the film were significantly passivated, leading to a suppressed non‐radiative recombination process in the modified perovskite film. Further, the addition of the IL EMIMCOOH significantly increased the average lifetime associated with carrier radiative recombination in the perovskite layer from 287.1 ns to 530.7 ns, as shown in Figure 4h and Figure S12 (Supporting Information). Using Equation (1) with a double‐exponential form, we modeled the lifetimes of carriers.

(1) $$I\left(t\right)=A_{1}e^{\left(-t/t_{1}\right)}+A_{2}e^{\left(-t/t_{1}\right)}+I_{0}$$

where *τ_1_* and *τ_2_* represent distinct carrier lifetimes associated with different recombination mechanisms. The calculated average lifetime *τ_ave_* was provided in Table S1 (Supporting Information). This extended lifetime indicated that EMIMCOOH enhanced the quality of perovskite films by reducing defects and minimizing nonradiative recombination and carrier quenching. To quantify the defects change in the perovskite layer, we conducted space‐charge‐limited current (SCLC) measurements on the hole‐only devices with ITO/PTAA/PVK/Spiro‐OMeTAD/Ag configurations under dark conditions. Figure 4i–j illustrated the *J*–*V* curves for both control and EMIMCOOH‐modified devices, showing the measured dark current density‐voltage relationship. The voltage at which the current rapidly increases was defined as the trap‐filling voltage (*V_TFL_*). Equation (2) expressed the correlation between trap density and trap filling voltage, as described by the Mott‐Gurney law.^[^ ^21^, ^62^ ^]^

(2) $$N_{trap}=\frac{2\varepsilon_{0}\varepsilon V_{TFL}}{eL^{2}}$$

where *ε_0_* is the dielectric constant of the vacuum, *ε* is the dielectric constant of the perovskite film, *e* represents the basic electronic charge, and *L* stands for the thickness of the perovskite film. The defect density of states was calculated by the formula S2, and the *N_trap_* values of *V_TFL_* and corresponding devices were shown in Table S2 (Supporting Information). The trap density of the EMIMCOOH‐modified perovskite film decreased from 4.08 × 10^15^ cm^−3^ to 3.75 × 10^15^ cm^−3^.

**Figure 5:**
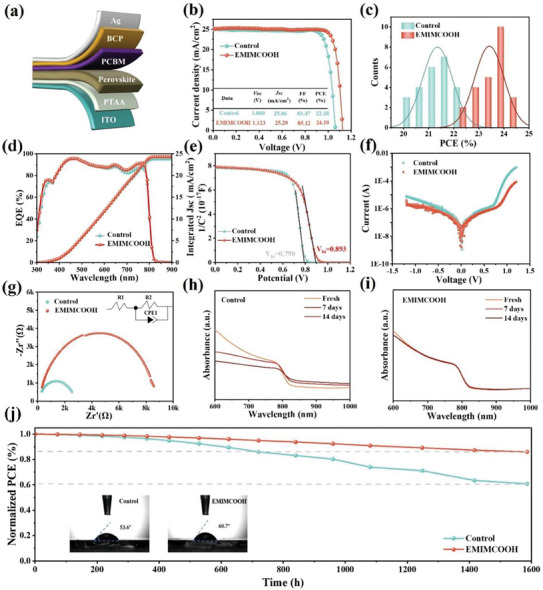
a) Schematic of the p‐i‐n PSC structure. b) *J–V* curves of the PSCs without or with EMIMCOOH. c) Statistical distributions of PCEs for the control device and the EMIMCOOH‐modified device, respectively. d) EQE spectra and integrated photocurrents of the control device and the device treated with EMIMCOOH. e) Mott‐Schottky plots, f) Dark *J–V* curves, and g) the Nyquist plots of the control device and the EMIMCOOH‐modified device, respectively. The UV–vis absorbance spectra of h) the control perovskite film and i) the perovskite film treated with EMIMCOOH in an air environment at 25 °C and 60% RH. j) PCE evolution of unencapsulated devices aged in an environment at 25 °C under a N_2_ atmosphere.

We incorporated IL EMIMCOOH into the PbI_2_ precursor solution and fabricated PSCs using a two‐step sequential deposition method, constructing a structure of ITO/PTAA/PVSK/PCBM/BCP/Ag as shown in **Figure** **5**a. The cross‐sectional SEM image of PSCs was shown in Figure S13 (Supporting Information). Figure 5b and Table S3 (Supporting Information) illustrated the *J–V* characteristics of the PSCs. The control device achieved a champion efficiency of 22.18%, *V_OC_* of 1.060 V, *J_SC_* of 25.06 mA cm^−2^, and *FF* of 83.47%. With EMIMCOOH, the champion device reached a PCE of 24.10%, *V_OC_* of 1.123 V, *J_SC_* of 25.20 mA cm^−2^, and an *FF* of 85.12%. Figure 5c presented a histogram illustrating the efficiency distribution for PSCs with and without EMIMCOOH treatment, along with statistical data for *V_OC_*, *J_SC_*, *FF*, and PCE as shown in Figure S14 (Supporting Information). The increase in *V_OC_* indicated that EMIMCOOH passivated the defects inside the perovskite crystals, suppressing the non‐radiative recombination process. It also contributed to improved charge transport properties with increased *FF* (over 85%). The measured external quantum efficiency (EQE) spectrum was shown in Figure 5d. The integrated *J_SC_* values from EQE curve of the control and IL EMIMCOOH‐modified the device were 23.92 mA cm^−2^ and 24.35 mA cm^−2^, respectively. These values aligned with the *J_SC_* measured by the *J–V* curve and fell within the error range of 5%. Additionally, we systematically investigated the impact of EMIMCOOH when the hole transport layers (HTLs) were replaced by other materials, such as 2PACz and 4PACz (Figures S15,S16 and Table S4, Supporting Information). The introduction of EMIMCOOH resulted in enhancement performance of inverted PSCs based on PTAA and the different HTLs, suggesting that our modification approach possesses broad applicability. Furthermore, a comparison of the results demonstrated that this modification strategy was significantly more effective in PTAA‐based devices. The Mott‐Schottky diagram (Figure 5e) indicated the built‐in voltage (*V_bi_*) increased from 0.790 V in the control device to 0.893 V with EMIMCOOH, suggesting a stronger ability to separate carriers and suppressed carrier recombination for EMIMCOOH‐modified perovskite layer.^[^ ^57^ ^]^ The *J*–*V* curve of the PSCs under dark conditions (Figure 4a) showed that the dark current of the EMIMCOOH‐modified device was significantly lower than that of the control device, indicating reduced leakage current and non‐radiative recombination for EMIMCOOH‐modified the device.^[^ ^63^ ^]^ The EIS curve (Figure 5g) illustrated that both devices exhibited a prominent semicircle at lower frequencies, attributed to the influence of the constant phase element and composite resistor (*R_rec_*).^[^ ^64^ ^]^ The IL EMIMCOOH‐modified the device had a more pronounced semicircle, indicating a significantly higher *R_rec_* and lower recombination rate compared to the control device.

To verify the impact of EMIMCOOH on the stability of the film, the UV–vis absorption spectra of control and EMIMCOOH‐modified perovskite film in an air environment at 25 °C and 60% relative humidity (RH) were tracked after 7 and 14 days, respectively, as shown in Figure 5h,i, the photographs of fresh and aged perovskite films are shown in Figure S17 (Supporting Information). The small attenuation was observed in EMIMCOOH‐modified perovskite film after 14 days of storage, indicating a significant enhancement in film stability. For long‐term stability, the devices were exposed to 25 °C under an N_2_ atmosphere for over 1500 h, with the normalized efficiency monitored.^[^ ^65^ ^]^ The EMIMCOOH‐modified device maintained 86% of its original PCE after more than 1500 h of aging, compared to 60.8% for the control. This enhanced stability was attributed to the higher quality of perovskite layer with decreased trap density and significantly reduced residual PbI_2_ clusters. The water contact angle of the EMIMCOOH‐modified perovskite thin film increased from 53.6° to 60.7°, implying enhanced hydrophobic properties and enhanced stability against humid conditions. It was also because perovskite devices containing EMIMCOOH can effectively passivate defects, reduce non‐radiative recombination losses caused by defects, and further improve the stability of the devices.

## 3 Conclusion

In summary, an ionic liquid engineering approach has been utilized to fabricate efficient and durable two‐step PTAA‐based p‐i‐n perovskite photovoltaic devices. By incorporating EMIMCOOH into the PbI_2_ precursor solution, a more pronounced mesoporous PbI_2_ film was obtained due to the coordination bond between the ─C═O group in EMIMCOOH and Pb^2+^. The intensified mesoporous PbI_2_ layer, with expended pore‐size and denser pores, facilitated the reaction between PbI_2_ and organic materials and promoted the complete conversion of PbI_2_ into perovskite material, leaving minor PbI_2_ residues. In situ characterizations revealed that the incorporation of the EMIMCOOH slowed down the perovskite conversion process, resulting in a uniform perovskite layer with increased domain‐size and suppressed the generation of Pb^0^ trap density. Moreover, the cation EMIM^+^ and the anion HCOO^‐^ of EMIMCOOH can interact with the under‐coordinated Pb^2+^, passivating the charge defects of perovskite, reducing non‐radiative recombination, and enhancing charge transport properties, as evidenced by the conducting AFM results. As a result, the EMIMCOOH IL modified device achieved an efficiency of 24.10% and a high fill factor of 85.12%. The device maintained 86% of its original PCE after more than 1500 h at 25 °C in a nitrogen environment without encapsulation. This work not only demonstrates the effectiveness of IL engineering in fabricating sufficient and durable PTAA based p‐i‐n perovskite photovoltaics, but also promotes the further application of ionic liquids in the photovoltaic industry.

## Conflict of Interest

The authors declare no conflict of interest.

## Supporting information



Supporting Information

## Data Availability

Research data are not shared.
