# Supplementary material for: PTAA‐Based Perovskite Photovoltaics Catching up: Ionic Liquid Engineering‐Assisted Crystallization Through Sequential Deposition
Source: Adv Sci (Weinh). 2025 Feb 20;12(15):2414515. doi: 10.1002/advs.202414515 (PMC12005749; doi:10.1002/advs.202414515)
Supplement: Supplementary file 1 — Supporting Information [file ADVS-12-2414515-s001.docx]

PTAA-Based Perovskite Photovoltaics Catching Up: Ionic Liquid Engineering-Assisted Crystallization through Sequential Deposition

Yongjun Li^1,2‡^, Fei Wang^1,3‡^, Qiannan Li^1‡^, Baolei Tang^1^*, Yonggui Sun^1^, Taomiao Wang^1^, Xiao Liang^1^, Jing Ma^4^*, Xianfang Zhou^1^, Fan Zhang^1,2^, Xing’ao Li^2^, Yao Tong^1^*, Ruiyuan Hu^2^*, Mingjian Yuan^5^, Tom Wu^6^, Annie Ng^7^*, Hanlin Hu^1^*.

^1^Hoffmann Institute of Advanced Materials, Shenzhen Polytechnic University, 7098 Liuxian Boulevard, Shenzhen 518055, China.

^2^Jiangsu Provincial Engineering Research Center of Low-Dimensional Physics and New Energy & School of Science, Key Laboratory for Organic Electronics and Information Displays & Institute of Advanced Materials (IAM), Jiangsu National Synergistic Innovation Center for Advanced Materials (SICAM), Nanjing University of Posts and Telecommunications, Nanjing 210023, China.

^3^State Key Laboratory of Advanced Technology for Materials Synthesis and Processing, School of Materials Science and Engineering, Wuhan University of Technology, Wuhan 430070, China.

^4^Medical Intelligence and Innovation Academy, Southern University of Science and Technology Hospital, 518055, China.

^5^College of Chemistry, Nankai University, Tianjin, China.

^6^Department of Applied Physics, The Hong Kong Polytechnic University, Kowloon, Hong Kong.

^7^Electrical and Computer Engineering, Nazarbayev University, 53 Kabanbay Batyr Avenue, Nur-Sultan, Kazakhstan.

*Corresponding Authors.

E-mail address: tangbl@szpu.edu.cn (B.T.)

majing@sustech-hospital.com (J.M.)

yao_tong@szpu.edu.cn (Y.T.)

ruiyuanhu@njupt.edu.cn (R.H.)

annie.ng@nu.edu.kz (A.N.)

hanlinhu@szpu.edu.cn (H. H.)

^‡^Both authors contributed equally to this work.

**Experimental Section**

*Materials:* ITO glass substrates with a sheet resistance of approximately 9 Ω sq^−1^ were purchased from OPVTECH Inc. Poly [bis(4-phenyl)(2,4,6-trimethylphenyl)amine] (PTAA, Mw ≈15,000, >99%), bathocuproine (BCP), Formamidinum Iodide (FAI), methylammonium iodide (MAI), and methylammonium chloride (MACL) were supplied by Xi'an Polymer Light Technology Corp. [6,6]-Phenyl-C61-butyric acid methyl ester (PCBM) was purchased from Nano-C Tech. PbI_2_ (99.8%) was supplied by Sigma-Aldrich. Ionic liquids 1-Ethyl-3-methylimidazolium formate (EMIMCOOH), chlorobenzene (CB), dimethylformamide (DMF), dimethyl sulfoxide (DMSO), and isopropanol (IPA) were purchased from Aladdin. All chemicals were used as received without any further treatment.

*Devices Fabrications:* To facilitate the device preparation, ITO substrates were sequentially cleansed using a sonication method, involving detergent, deionized (DI) water, acetone, and isopropanol for 30 minutes each. Subsequently, the purified ITO substrates underwent a 15-minute treatment with ultraviolet ozone.

For the devices discussed in this paper, a two-step sequential deposition method will be employed. The substrates were transferred into a glove box under a nitrogen atmosphere. Subsequently, the PTAA solution (2.5 mg/ mL in chlorobenzene) was spin-coated onto the ITO glass substrate at 5000 rpm for 30 seconds, followed by annealing at 100°C for 10 minutes. When it cools to room temperature, the perovskite layer is fabricated using a modified two-step sequential process within the glovebox. Firstly, 1.5M of PbI_2_ with or without ILs EMIMCOOH was added to a DMF: DMSO (9:1) solvent mixture, which was spin-coated onto PTAA at 1500 rpm for 30 s with a ramping rate of 1000 rpm/s. The film was then annealed at 70 °C for 1 min and left to cool down to room temperature for two to three minutes. A solution containing FAI: MAI: MACL (90 mg: 6.4 mg: 9 mg in 1 mL IPA) was spin-coated onto the PbI_2_ layers at a speed of 2000 rpm for 30 seconds with a ramping rate of 3000 rpm/s, followed by annealing at 150°C for 15 minutes. Afterward, the perovskite layer was sequentially spin-coated with PCBM (20 mg/mL in chlorobenzene) and BCP (1 mg/mL in isopropanol) at 2000 and 5000 rpm, respectively. Finally, a 100 nm Ag electrode was deposited using thermal evaporation.

*Characterization:* GIWAXS measurements were conducted at the Synchrotron and Printable Electronics Laboratory at Shenzhen Polytechnic using Saxs Focus, which was equipped with a Cu X-ray source (8.05 keV, 1.54 Å) and a Pilatus3R 300K detector. The incident angle was set to 0.5°. X-ray diffraction spectra of the perovskite film were obtained by scanning angles ranging from 3 to 40° (2θ) using a Bruker D8 Advance instrument. The current-voltage characteristics of the PSC devices were evaluated using an IVS-KA6000 Enlitech sunshine simulator equipped with an AM1.5 filter under an illumination intensity of 100 mWcm^-2^ and controlled by a Keithley SMU source following calibration against a standard reference cell. The J-V curves were acquired through forward scans (-0.2 to 1.2 V) followed by reverse scans (1.2 to -0.2 V). An QE-R device manufactured by Enli Technology Co. Ltd. The instrument was used to obtain the corresponding External Quantum Efficiency (EQE) spectrum under ambient conditions. Scanning electron microscopy (SEM) studies were conducted using a JSM-IT800 to observe the morphology of a perovskite thin film. Atomic force microscope (AFM) characterizations were conducted using an Oxford Instruments MFP-3D Origin. The Edinburgh FLSP1000 spectrophotometer, equipped with a 440 nm picosecond pulsed diode laser excitation source, was used to measure the static photoluminescence (PL) and time-resolved photoluminescence (TRPL) of the perovskite film.


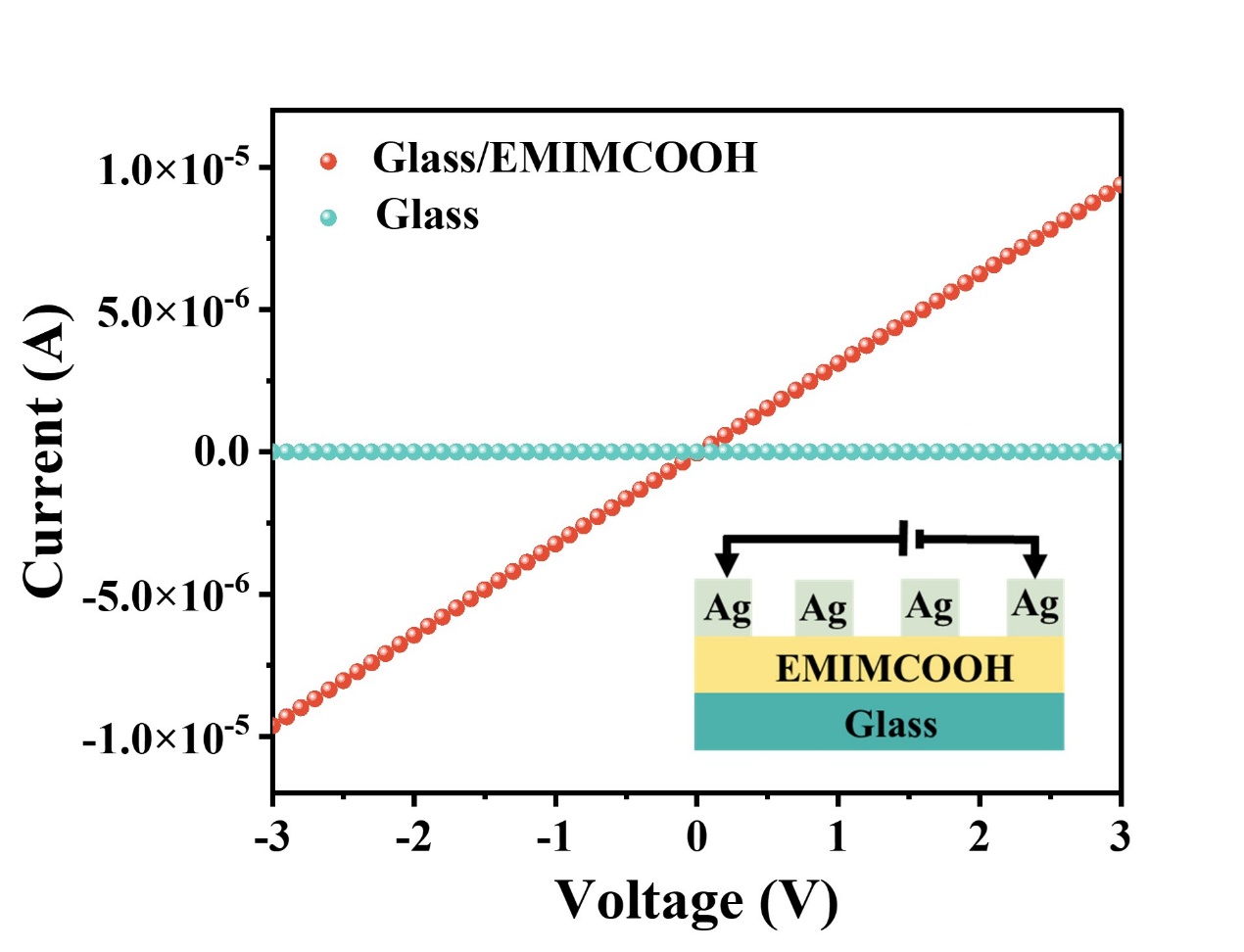


**Figure S1**. *I−V* characteristic curves of samples with structure of Glass/EMIMCOOH/Ag and Glass/Ag.


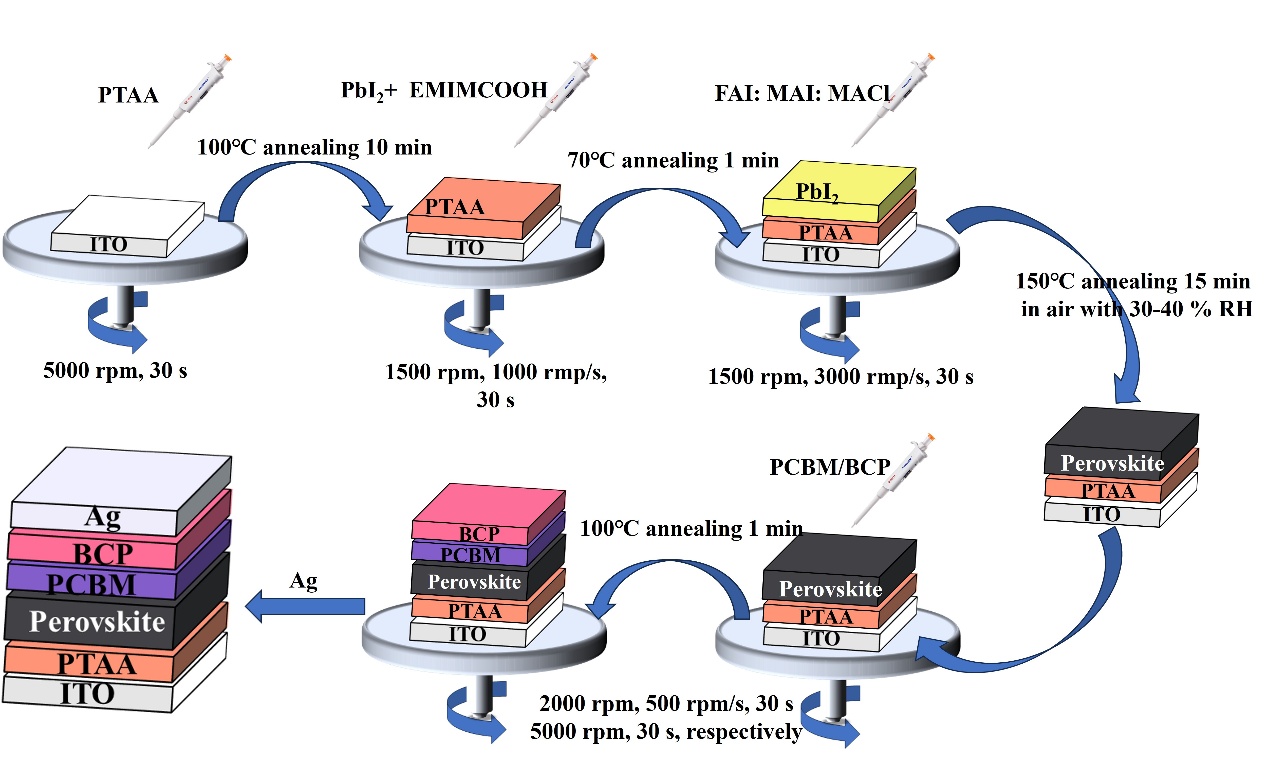


**Figure S2.** Schematic diagram of fabrication procedure of PSCs.


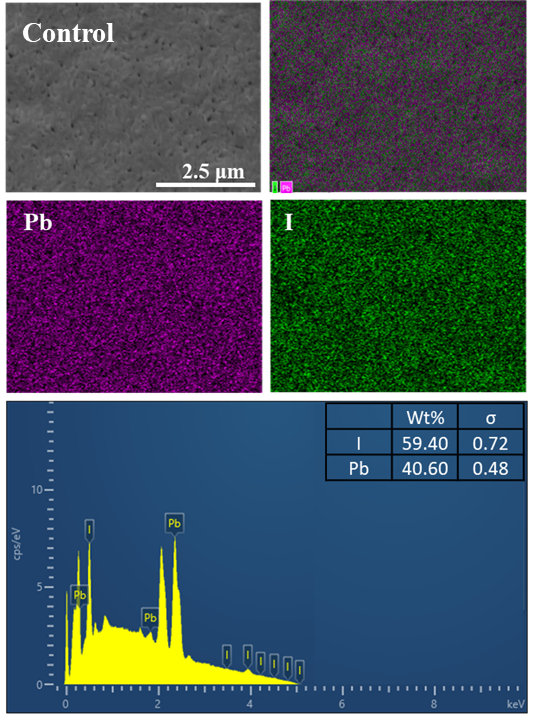


**Figure S3.** EDS mapping of control PbI_2_ film and statistics of corresponding elements content.


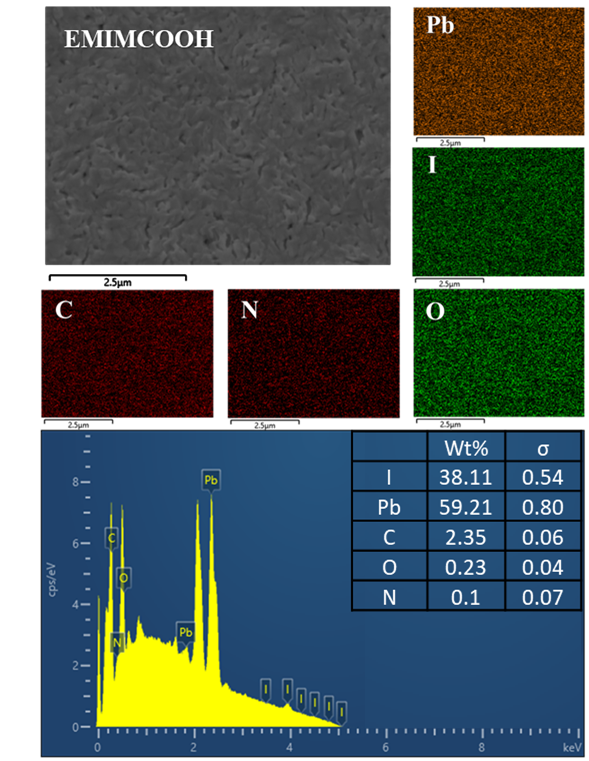


**Figure S4.** EDS mapping of EMIMCOOH-modified PbI_2_ film and statistics of corresponding elements content.


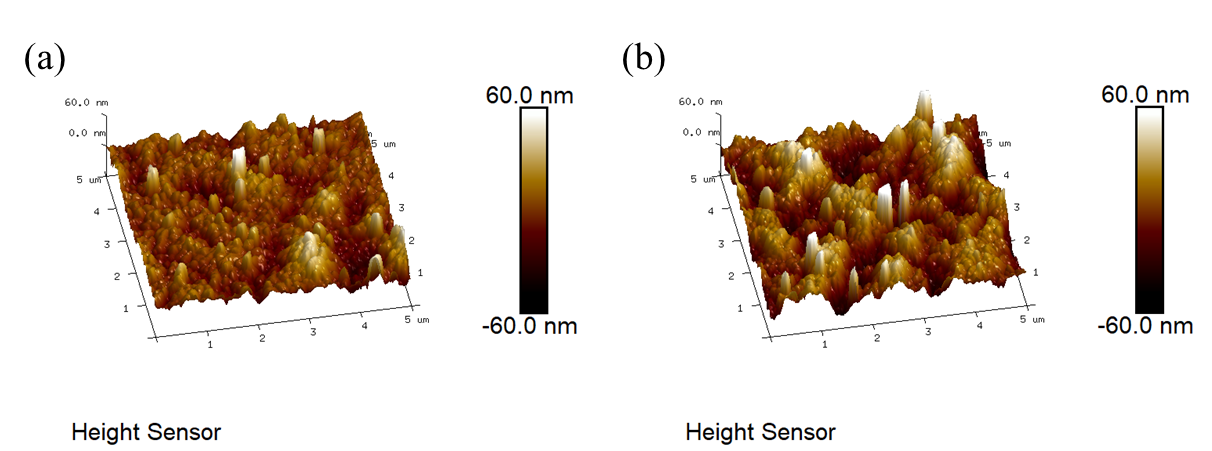


**Figure S5**. AFM images of (a) control and (b) EMIMCOOH-modified PbI_2_ films.


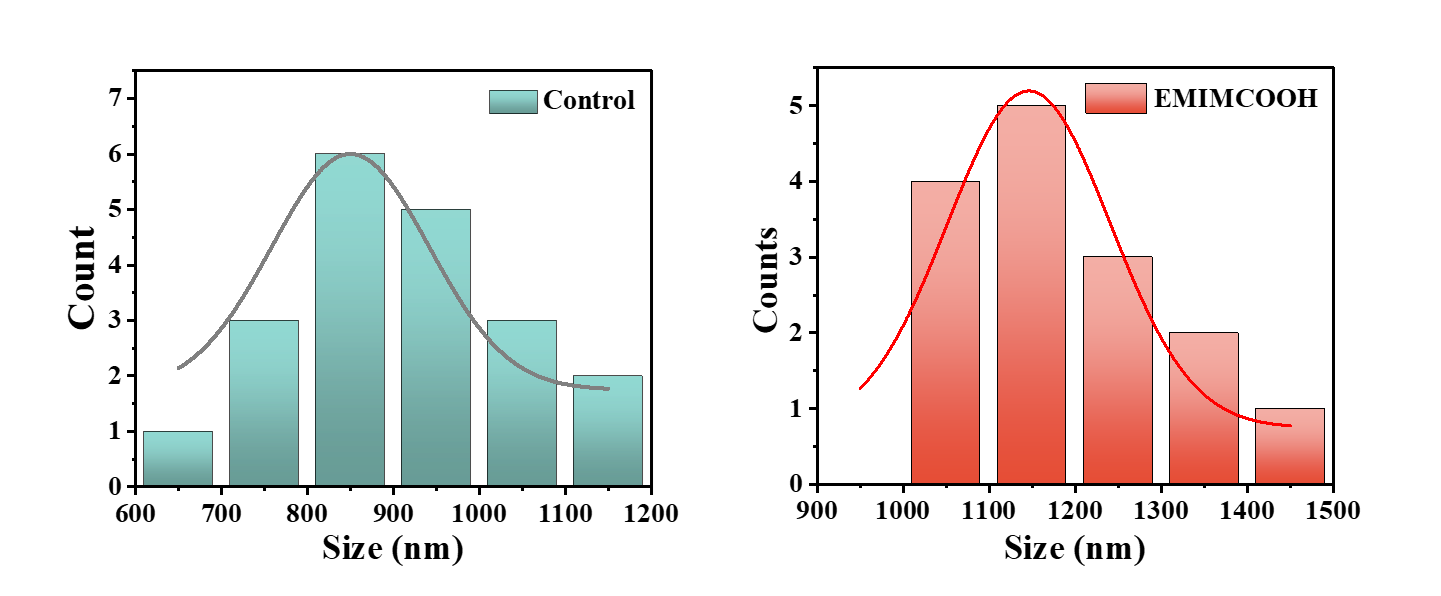


**Figure S6.** The grain size distribution of the control and EMIMCOOH-modified perovskite films.


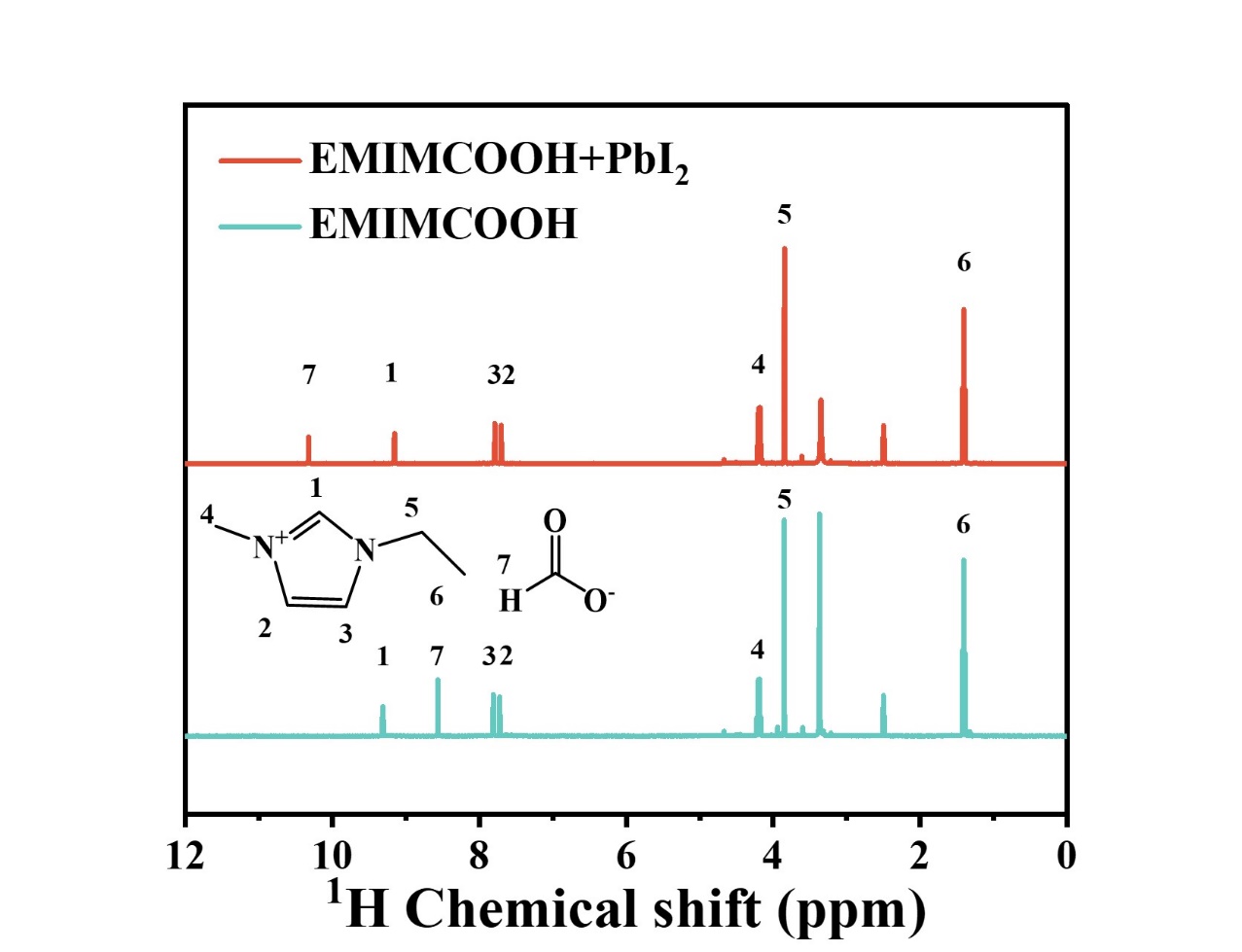


**Figure S7.** ^1^H NMR spectra of the EMIMCOOH and EMIMCOOH+PbI_2_ samples.


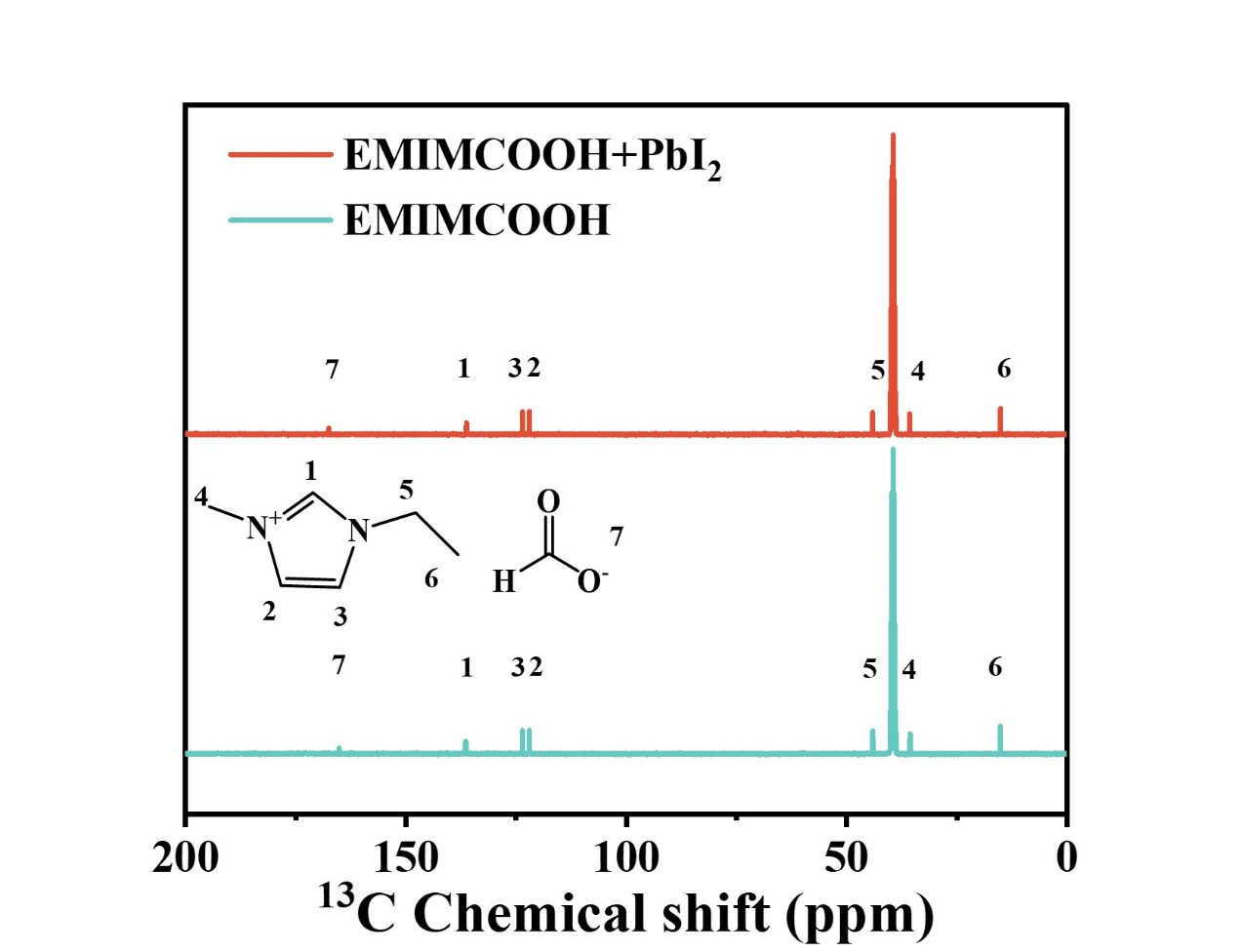


**Figure S8.** ^13^C NMR spectra of the EMIMCOOH and EMIMCOOH+PbI_2_ samples.


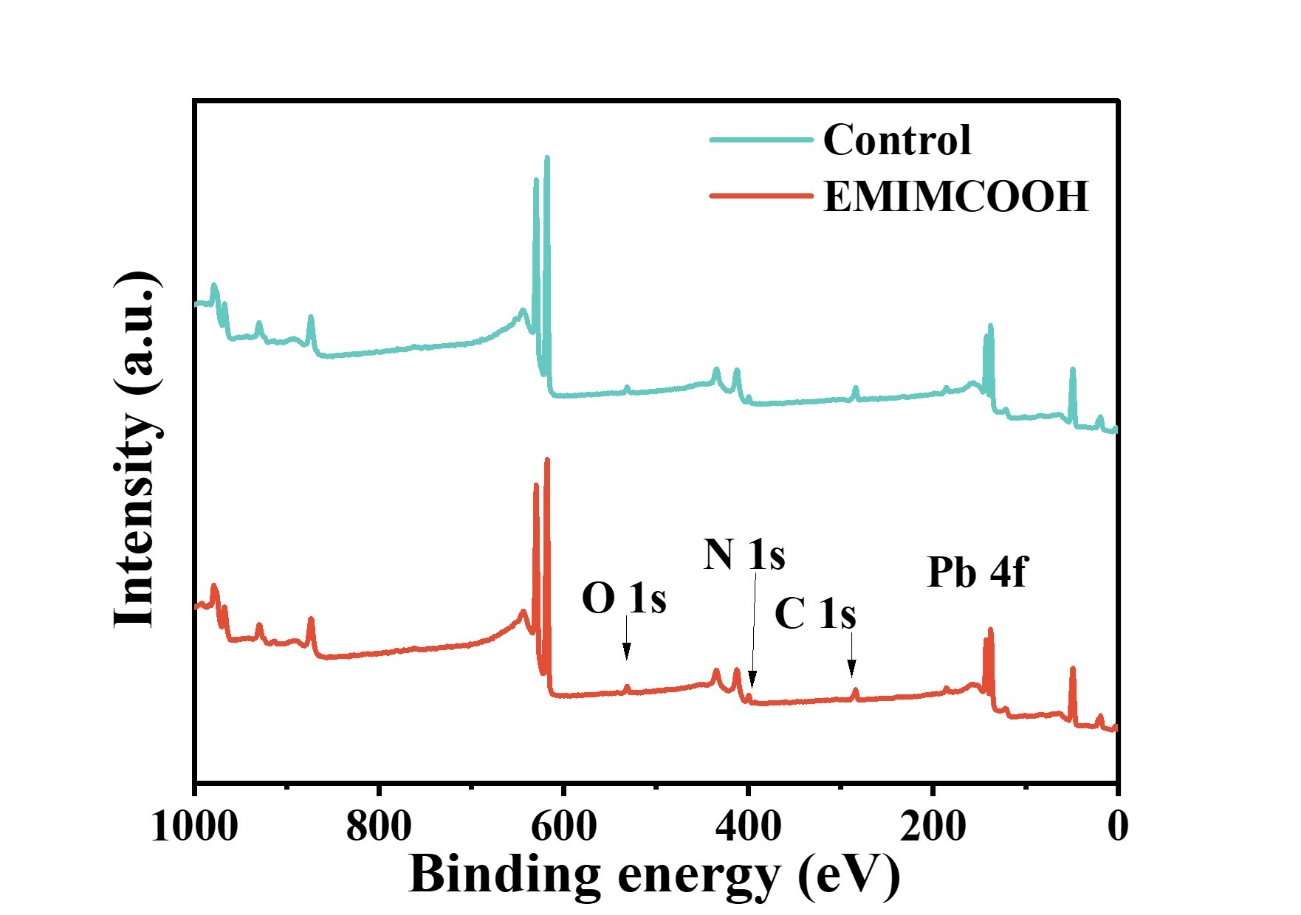


**Figure S9.** XPS spectra of control perovskite thin film and EMIMCOOH-modified perovskite thin film.

**Figure S10.** XRD pattern of the perovskite films with different concentrations of the EMIMCOOH.

**
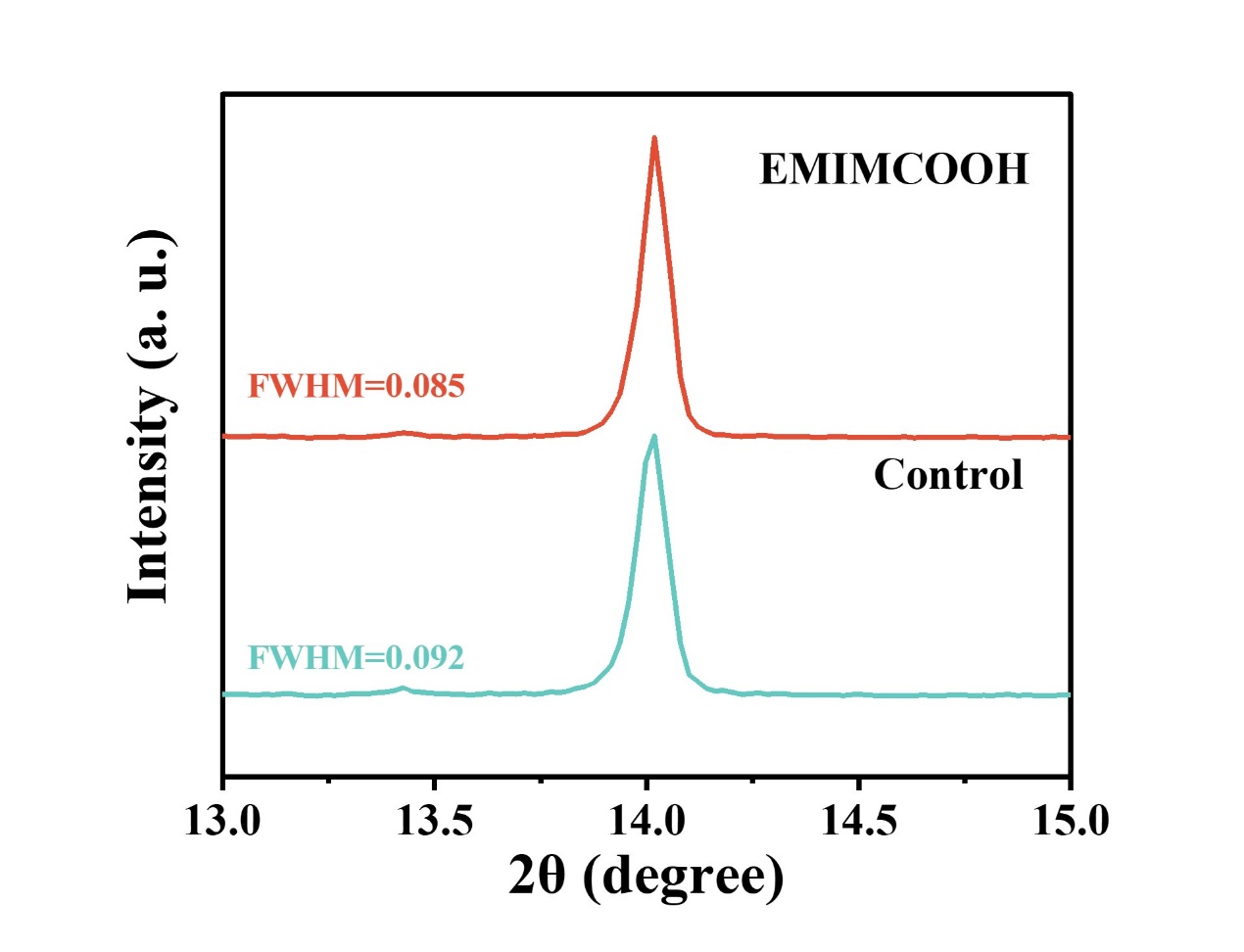
**

**Figure S11.** FWHM of the control and EMIMCOOH-modified perovskite film


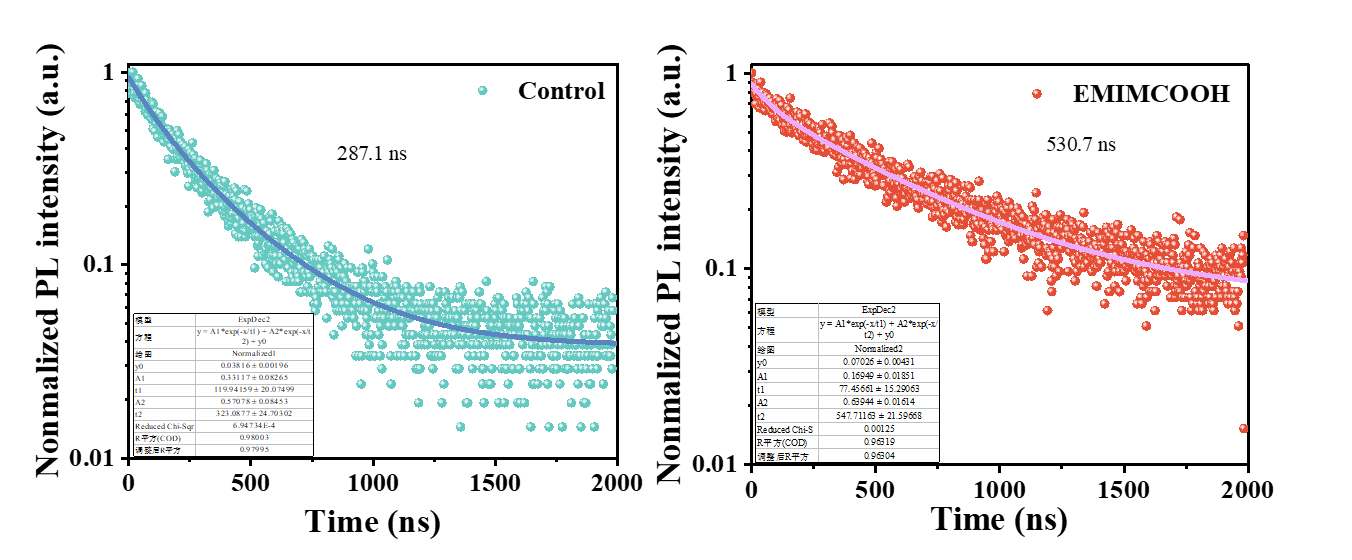


**Figure S12.** Time-resolved photoluminescence spectra of the control and EMIMCOOH-modified perovskite films.


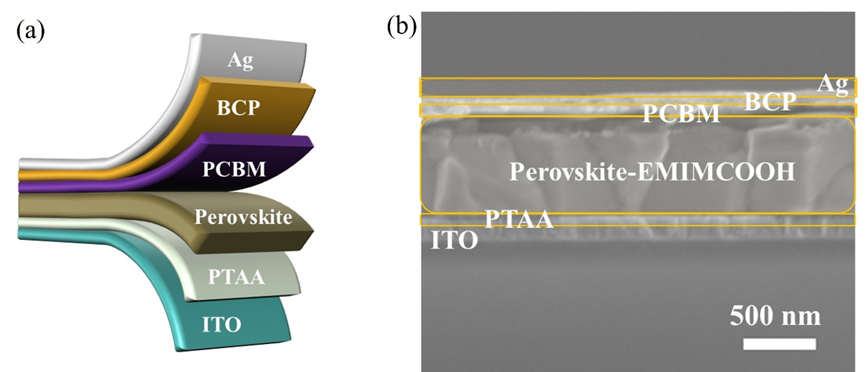


**Figure S13.** (a) The schematic illustration of the p-i-n structure. (b) The cross-sectional SEM images of the prepared PSCs with EMIMCOOH with a device structure of ITO/PTAA/perovskite/PCBM/BCP/Ag.


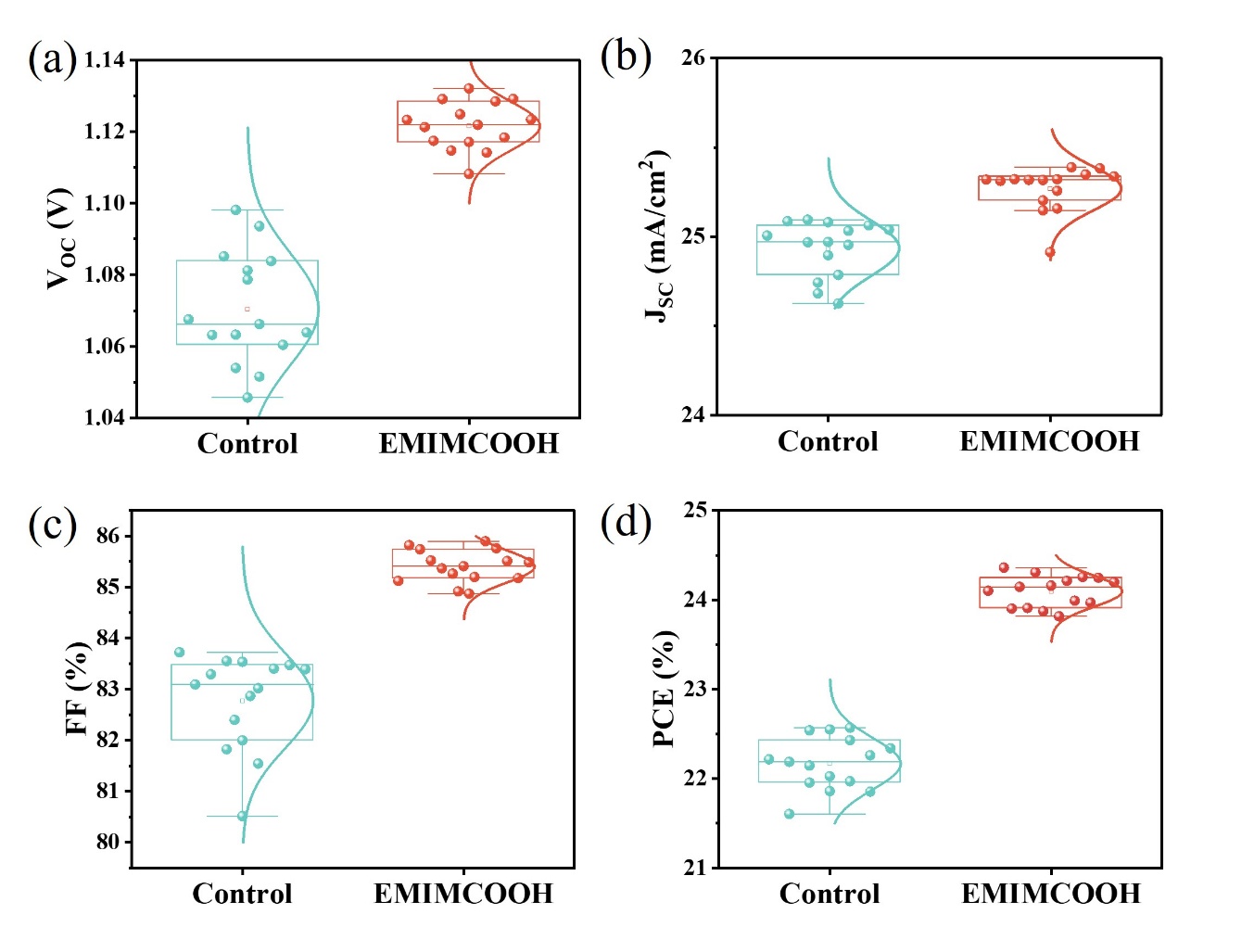


**Figure S14.** The statistics of photovoltaic performance parameters of control and EMIMCOOH-modified PSCs: (a) *V_OC_*, (b) *J_SC_*, (c) *FF* and (d) PCE.


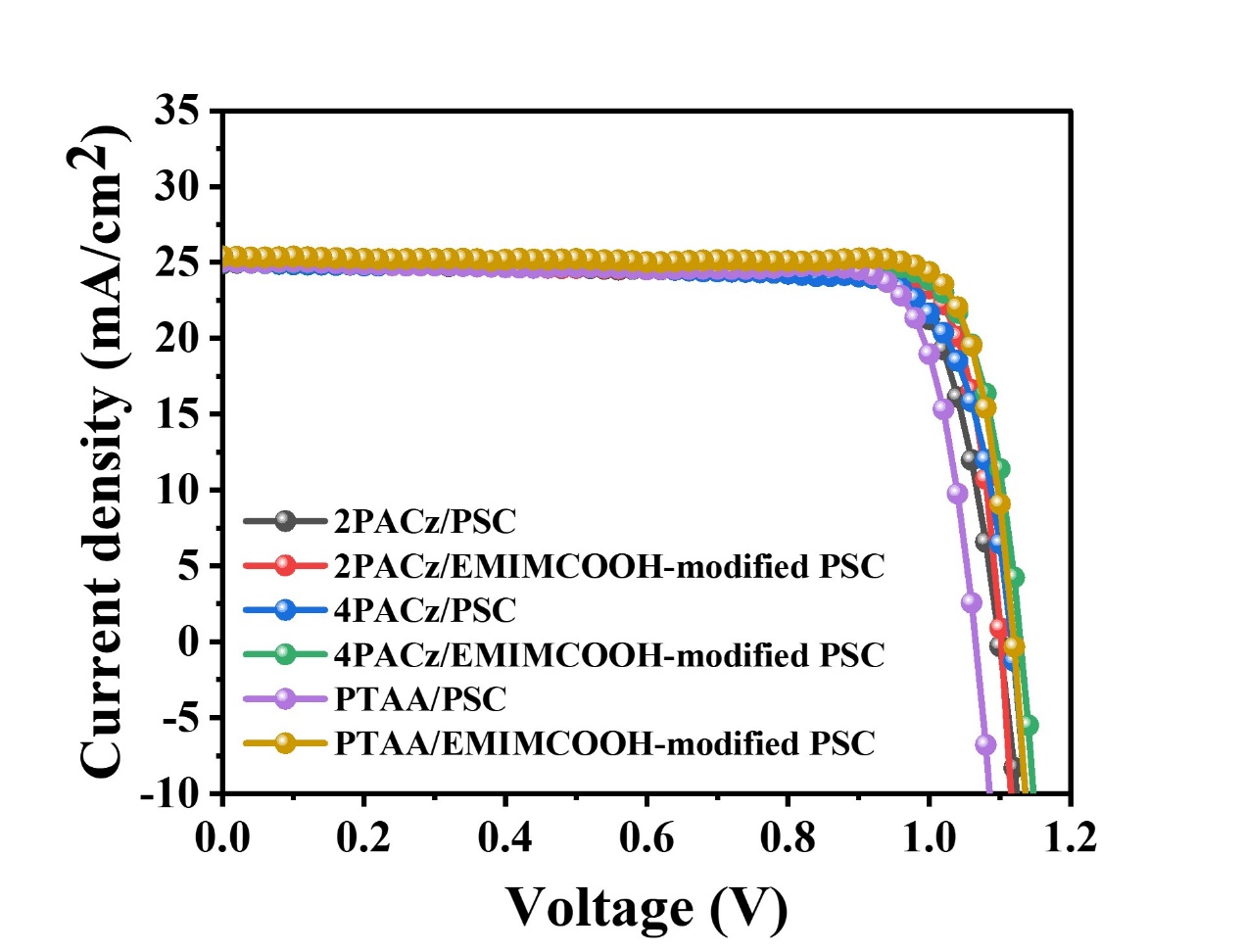


**Figure S15**. *J-V* curves of different hole transport layers of the PSCs without or with EMIMCOOH


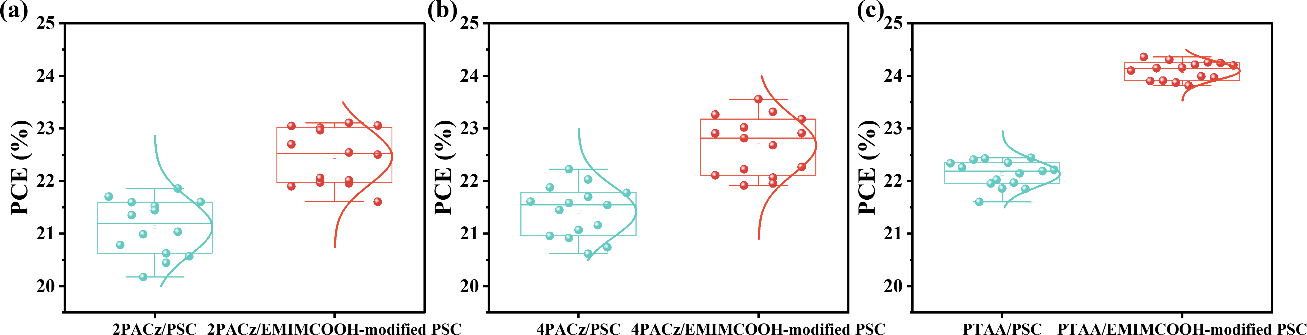


**Figure S16**. Statistical distributions of PCEs for different hole transport layers of the PSCs without or with EMIMCOOH.


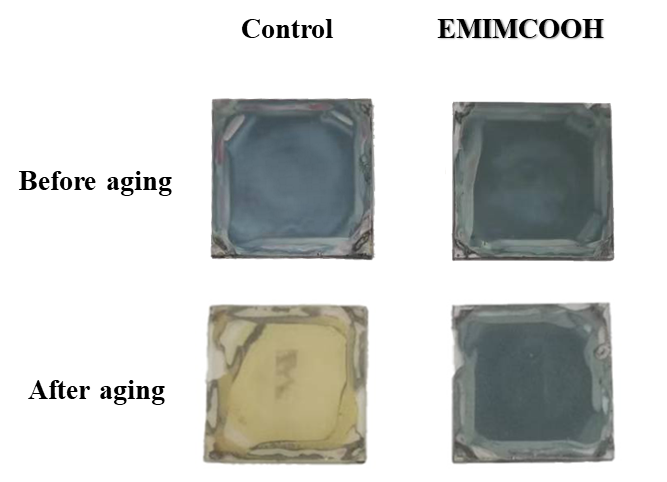


**Figure S17**. The before aging and after aging of control and EMIMCOOH-modified perovskite films at 25 °C, 60 % RH in air environment, respectively.

**Table S1.** Detailed TRPL parameters of control and EMIMCOOH-modified perovskite films.

| Conditions | *τ_1_* (ns) | *A_1_* (%) | *τ_2_* (ns) | *A_2_* (%) | *τ_ave_* (ns) |
| --- | --- | --- | --- | --- | --- |
| Control | 119.9 | 33.1 | 323.0 | 57.0 | 287.1 |
| EMIMCOOH | 77.4 | 16.9 | 547.7 | 63.9 | 530.7 |

$\tau_{ave}$ = (${A_{1}\tau}_{1}^{2}+A_{2}\tau_{2}^{2}$)/($A_{1}\tau_{1}+A_{2}\tau_{2}$)

**Table S2.** The *V*_TFL_ and calculated *N*_trap_ values of the control and EMIMCOOH modified hole-only devices calculated by SCLC method.

|  | *V_TFL_* (V) | *N_trap_* (cm^-3^) |
| --- | --- | --- |
| Control | 0.739 | 4.08×10^15^ |
| EMIMCOOH | 0.679 | 3.75×10^15^ |

**Table S3.** Photovoltaic parameters of the control and EMIMCOOH-modified devices.

|  | *J_SC_* (mA/cm^2^) | *V_OC_* (V) | *FF* (%) | PCE (%) |
| --- | --- | --- | --- | --- |
| Control | 25.06 | 1.060 | 83.47 | 22.18 |
| EMIMCOOH | 25.20 | 1.123 | 85.12 | 24.10 |

**Table S4**. Photovoltaic parameters of different hole transport layers of the PSCs without or with EMIMCOOH.

|  | *J_SC_* (mA/cm^2^) | *V_OC_* (V) | *FF* (%) | PCE (%) |
| --- | --- | --- | --- | --- |
| 2PACz/PSC | 24.95 | 1.098 | 81.54 | 22.34 |
| 2PACz/EMIMCOOH-modified PSC | 25.14 | 1.099 | 84.25 | 23.28 |
| 4PACz/PSC | 24.85 | 1.114 | 80.52 | 22.29 |
| 4PACz/EMIMCOOH-modified PSC | 24.91 | 1.127 | 84.25 | 23.51 |
| PTAA/PSC | 25.04 | 1.064 | 83.39 | 22.22 |
| PTAA/EMIMCOOH-modified PSC | 25.38 | 1.117 | 85.51 | 24.26 |
